# Enroll-HD: An Integrated Clinical Research Platform and Worldwide Observational Study for Huntington's Disease

**DOI:** 10.3389/fneur.2021.667420

**Published:** 2021-08-18

**Authors:** Swati Sathe, Jen Ware, Jamie Levey, Eileen Neacy, Robi Blumenstein, Simon Noble, Alzbeta Mühlbäck, Anne Rosser, G. Bernhard Landwehrmeyer, Cristina Sampaio

**Affiliations:** ^1^CHDI Management/CHDI Foundation, Princeton, NJ, United States; ^2^Department of Neurology, Ulm University, Ulm, Germany; ^3^Brain Repair Group, School of Biosciences, Cardiff University, Cardiff, United Kingdom; ^4^Division of Psychological Medicine and Clinical Neurosciences, MRC Centre for Neuropsychiatric Genetics and Genomics, School of Medicine, Cardiff University, Cardiff, United Kingdom; ^5^Brain Research and Intracranial Neurotherapeutics Unit, Cardiff University, Cardiff, United Kingdom

**Keywords:** Huntington's disease, disease network, registry, clinical research platform, longitudinal observational cohort study, Enroll-HD

## Abstract

Established in July 2012, Enroll-HD is both an integrated clinical research platform and a worldwide observational study designed to meet the clinical research requirements necessary to develop therapeutics for Huntington's disease (HD). The platform offers participants a low-burden entry into HD research, providing a large, well-characterized, research-engaged cohort with associated clinical data and biosamples that facilitates recruitment into interventional trials and other research studies. Additional studies that use Enroll-HD data and/or biosamples are built into the platform to further research on biomarkers and outcome measures. Enroll-HD is now operating worldwide in 21 countries at 159 clinical sites across four continents—Europe, North America, Latin America, and Australasia—and has recruited almost 25,000 participants, generating a large, rich clinical database with associated biosamples to expedite HD research; any researcher at a verifiable research organization can access the clinical datasets and biosamples from Enroll-HD and nested studies. Important operational features of Enroll-HD include a strong emphasis on standardization, data quality, and protecting participant identity, a single worldwide study protocol, a flexible EDC system capable of integrating multiple studies, a comprehensive monitoring infrastructure, an online portal to train and certify site personnel, and standardized study documents including informed consent forms and contractual agreements.

## Introduction

Enroll-HD is a worldwide integrated clinical research platform that has, at its core, an observational study that has recruited almost 25,000 participants. In concert, the platform and study are designed to meet the clinical research requirements necessary to successfully develop and evaluate therapeutics for Huntington's disease (HD) (1). Enroll-HD is supported—financially, scientifically, and managerially—by CHDI Foundation, a nonprofit biomedical research organization solely dedicated to collaboratively developing therapeutics that will substantially improve the lives of those affected by HD. Here, we outline the Enroll-HD platform's objectives, scope, operational infrastructure, associated research studies, clinical trial recruitment and site feasibility services, advisory/educational outreach, and governance structure, and provide an overview of the Enroll-HD observational study, including the cohort dataset and associated biosamples and how any researcher can access these resources.

HD is a rare, adult-onset, autosomally-dominant neurodegenerative disorder caused by the dynamic expansion of a polymorphic CAG repeat in exon 1 of the *huntingtin* (*HTT*) gene that encodes the mutant huntingtin (HTT) protein (2), with an estimated prevalence of 5–17 individuals per 100,000 (3, 4). CAG-repeat length is inversely related to age of onset; a CAG-repeat length between 8 and 26 is normal, whereas CAG-repeat lengths of 40 or more are fully penetrant. CAG repeats of 36–39 have reduced penetrance, and CAG-repeat lengths of 27–35 are considered intermediate alleles that do not cause pathology in the carrier but could expand to pathogenic length after transmission through the germline (5).

Clinically, HD is a prototypic monogenic neurodegenerative disease with a protracted but relentlessly progressive course. Although fluctuating and often worsening psychological manifestations are notable early on (6, 7), progressive involuntary movement disorder (predominantly chorea) is the definitive manifestation, with a simultaneous decline in cognitive function that, together, lead to severe morbidity, disability and, ultimately, death (8, 9). Classically, the appearance of motor dysfunction defines the clinical onset of disease, usually termed “motor onset” or “motor diagnosis.” The CAP score (CAG-Age-Product, i.e., the product of excess CAG length and age) is a commonly used predictor of disease states in HD, including motor onset, and a reference for disease progression statistics (10). Currently, there are no disease-modifying therapeutics for HD but extensive ongoing research into HTT-lowering agents holds promise despite recent trial setbacks (11), as do gene-therapy approaches to silence the mutant gene.

## Enroll-HD Platform

**Figure 1:**
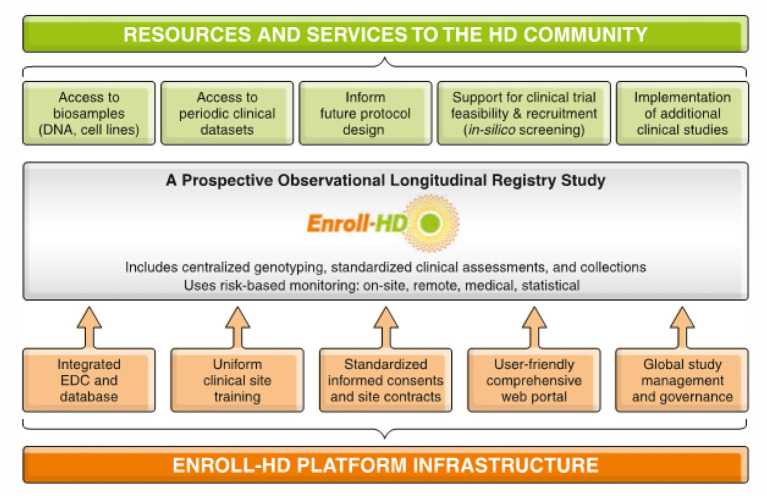
Schematic outlining the Enroll-HD clinical research platform.

The Enroll-HD platform has been designed with the benefit of experience gained from several foundational HD studies—REGISTRY, PREDICT-HD, COHORT, and TRACK-HD/Track On-HD (12–16). Enroll-HD's main objectives are to improve the design and expedite the recruitment and execution of clinical trials and studies, improve our understanding of HD and the factors that influence disease progression, and promote good clinical care to improve the health of individuals with HD (Figure 1 and Table 1). The platform was designed as a low-burden entry for participants into HD research, and provides a large, well-characterized, research-engaged cohort with associated clinical data and biosamples to facilitate recruitment into interventional trials and other research studies. The platform infrastructure and HD-expert network is available to trial/study sponsors from industry and academia to prospectively assess trial/study-design feasibility, identify clinical sites with eligible participants and appropriate resources, and assist with key operational aspects.

**Table 1:** Objectives of the Enroll-HD observational study.

**1. To provide a platform to support the design and conduct of hypothesis-testing clinical trials and studies by:**
•identifying and developing novel assessment tools, clinical endpoints, and biomarkers, •collecting longitudinal participant data to inform disease modeling studies, •using “run-in” data to estimate rates of disease progression and inform the selection of potential trial participants.
**2. To improve the understanding of the dynamic phenotypic spectrum and the pathobiological mechanisms of HD by:**
•collecting observational data covering the cognitive, behavioral, and motor domains to estimate rates of progression and give insight into the neurobiology of HD, •collecting data and biosamples to identify genetic and environmental factors that can alter disease progression and the HD phenotype, •promoting exploratory studies that can give further insight into HD pathogenesis.
**3. To promote the development of evidence-based guidelines to inform clinical decision making and improve health outcomes for those affected by HD by:**
•identifying beneficial interventions (clinical, pharmaco-therapeutic, non-pharmacologic), •facilitating the dissemination and implementation of current best clinical practice, •providing a platform to conduct outcome research, •promoting exploratory data analysis projects to identify processes to improve the healthcare of affected individuals and their families.

## Enroll-HD Observational Study

Central to the clinical research platform is the Enroll-HD study (NCT01574053), a prospective longitudinal observational study that collects natural history data in HD gene-expansion carriers (HDGECs) and non-HDGEC controls. Established in July 2012, the Enroll-HD study has generated a large and rich clinical database with associated biosamples to support research, including developing disease-progression and prognostic biomarkers, identifying clinically relevant phenotypic characteristics, and establishing endpoints for interventional trials. As of January 1, 2021, Enroll-HD is operating worldwide in 21 countries at 159 sites across four continents—Europe, North America, South America, and Australasia—and has recruited 24,854 participants, 20,159 of whom are still currently enrolled, and 19,311 are currently active.

Two HD observational studies, REGISTRY and COHORT, were precursors to Enroll-HD. REGISTRY, a longitudinal observational cohort study conducted in Europe between 2004 and 2017 with >14,000 participants, was the result of strong collaboration and trusted partnership among preclinical and clinical investigators, patients and families in forming the European Huntington's Disease Network (EHDN). Enroll-HD has recruited 6,247 participants from REGISTRY who reconsented to continue participation and transfer their data.

Currently there is an emphasis on recruiting premanifest HDGEC participants into Enroll-HD, especially younger adults, to identify early biological and clinical characteristics with a view to designing clinical trials earlier in the disease course. Planning is now ongoing for a digital study, SelfEnroll, that will allow remote data collection and encourage younger participants to join.

## Patient Recruitment and Informed Consent

Participants are recruited at specialized HD clinics following a written informed consent that includes research genotyping. Donating biosamples (venous blood) for banking purposes, pedigree charting and family history, linking of data collected across other studies, and willingness to be contacted to consider participation in future trials/studies are additional optional components that require specific participant consent.

## Study Population

The study population comprises HDGECs (CAG expansion ≥36 on the longer allele) classified as:

1. Manifest HD: HDGECs age 18 or older who are deemed to have diagnostic HD clinical features in the opinion of the site investigator (and confirmed at each subsequent visit).
2. Premanifest HD: HDGECs age 18 or older who are deemed not to have diagnostic clinical features of HD.
3. Juvenile HDGECs: HDGECs under the age of 18 years who are clinically diagnosed with juvenile HD.

The control population comprises individuals who do not carry the *HTT* gene expansion (CAG expansion <36 on the longer allele) and includes three categories:

1. Genotype negative: first- or second-degree relative of an HDGEC, who has undergone predictive testing and does not have the CAG expansion.
2. Family control: family member or other individual not genetically related to an Enroll-HD HDGEC participant (e.g., spouses, partners, and caregivers).
3. Community control: individual not genetically related to an HDGEC, who did not grow up in an HD-affected family and does not have a concurrent neurological disorder.

Individuals from known HD families who are at risk of inheriting the *HTT* CAG expansion but do not wish to know their genetic status can enter the study as “genotype unknown” (see below).

## Enroll-HD Clinical Data and Biosample Collection

At their annual study visit, each participant is required to complete a core battery of assessments; extended and optional assessments are completed at the discretion of the PI and participant, respectively (Table 2). Visits vary between 45 min (core battery only) and 2.5 h (core battery plus extended and optional assessments), and data are collected regarding demographics, medical history (including comorbidity and pharmacotherapy), and clinical assessment of four HD domains—motor, cognition, behavior, and function. The Unified Huntington's Disease Rating Scale (UHDRS) is a widely used standardized clinical assessment that has been extensively evaluated for reliability and internal consistency (17). The UHDRS motor and diagnostic confidence index subscales characterize the clinical HD motor phenotype and capture the rater's diagnostic confidence regarding HD motor onset in participants, respectively. Total Functional Capacity, Functional Assessment and Independence Subscales of the UHDRS ‘99 assess participants' functional status, and cognition is assessed using the Categorical Verbal Fluency Test, Symbol Digit Modality Test, and Stroop Color, and Word Reading Test.

**Table 2:** Enroll-HD data elements and assessments.

**Data element/assessment**	**Core^**a**^**	**Extended^**b**^**	**Optional^**c**^**
**General**			
Investigator determined classification of participant	X		
Sociodemographic data	X		
HD clinical characteristics	X		
Medical history	X		
Comorbid conditions	X		
Current therapies (pharmacotherapies, non-pharmacologic therapies, and nutritional supplements)	X		
Reportable event monitoring	X		
**Motor**			
UHDRS '99 Motor	X		
UHDRS '99 Diagnostic Confidence Index	X		
Timed Up and Go		X	
30 second Chair Stand Test		X	
**Function**			
UHDRS '99 Total Functional Capacity	X		
UHDRS '99 Function Assessment Scale	X		
UHDRS '99 Independence Scale	X		
**Behavior**			
Problem Behaviors Assessment (Short)	X		
Hospital Anxiety and Depression Scale		X	
Snaith Irritability Scale		X	
Columbia Suicide Severity Rating Scale		X	
**Cognition**			
Symbol Digit Modality Test	X		
Stroop Word Reading Test	X		
Verbal Fluency Test (Category)	X		
Stroop Color Naming Test	X		
Stroop Interference Test		X	
Trail Making Tests (Parts A and B)		X	
Verbal Fluency Test (Letters)		X	
Mini Mental State Examination		X	
**Global assessment**			
Clinical Global Impression		X	
**Quality of Life**			
Short Form Health Survey 12v2		X	
Caregivers Quality of Life Questionnaire		X	
**Health Economics**			
Client Services Receipt Inventory		X	
Work Productivity and Activity Impairment-Specific Health Problem		X	
**Genotyping**			
Research CAG genotyping	X		
Local CAG genotyping (predictive/diagnostic) (if applicable)			
**Family history**			X
**Biosample donation**			X

a *Completed or updated at each annual visit*.

b *Completed at the discretion of the principal investigator*.

c *Completed at the discretion of the participant*.

Identity is protected by assigning an HDID code to each participant, generated *via* a secure system; no personally identifying information is stored in the EDC system, and a participant's data from another study can be linked using their HDID. Additionally, all participants are assigned both a lab ID and a research ID for use by the biorepository and other service providers (such as travel reimbursement), respectively, to avoid widespread sharing of their HDID. Similarly, a recoded participant ID, not the HDID, is used in publicly available dataset releases to further reduce risk of identification.

Research CAG genotyping is conducted at a central laboratory in Italy for every participant following their baseline visit, including those designated as “genotype unknown.” The CAG-repeat length defined in this research genotyping is used for all data analysis but is not reported back to the site investigator or participant. Participants who enroll as “genotype unknown” are reassigned to the appropriate HDGEC or genotype negative category at the time of data release under the recoded participant ID; the genetic status of these participants is not linked to their HDID as an extra protection. Data on reportable events—suicide attempts, completed suicide, mental health events requiring hospitalization, death from any other cause—are also captured. Most participants consent to donate biosamples; family history (pedigree) may also be recorded, subject to participant consent. The Data Dictionary and annotated eCRF at https://enroll-hd.org/for-researchers/technical-support/ contain a complete list of variables.

## Enroll-HD Study Cohort

**Figure 2:**
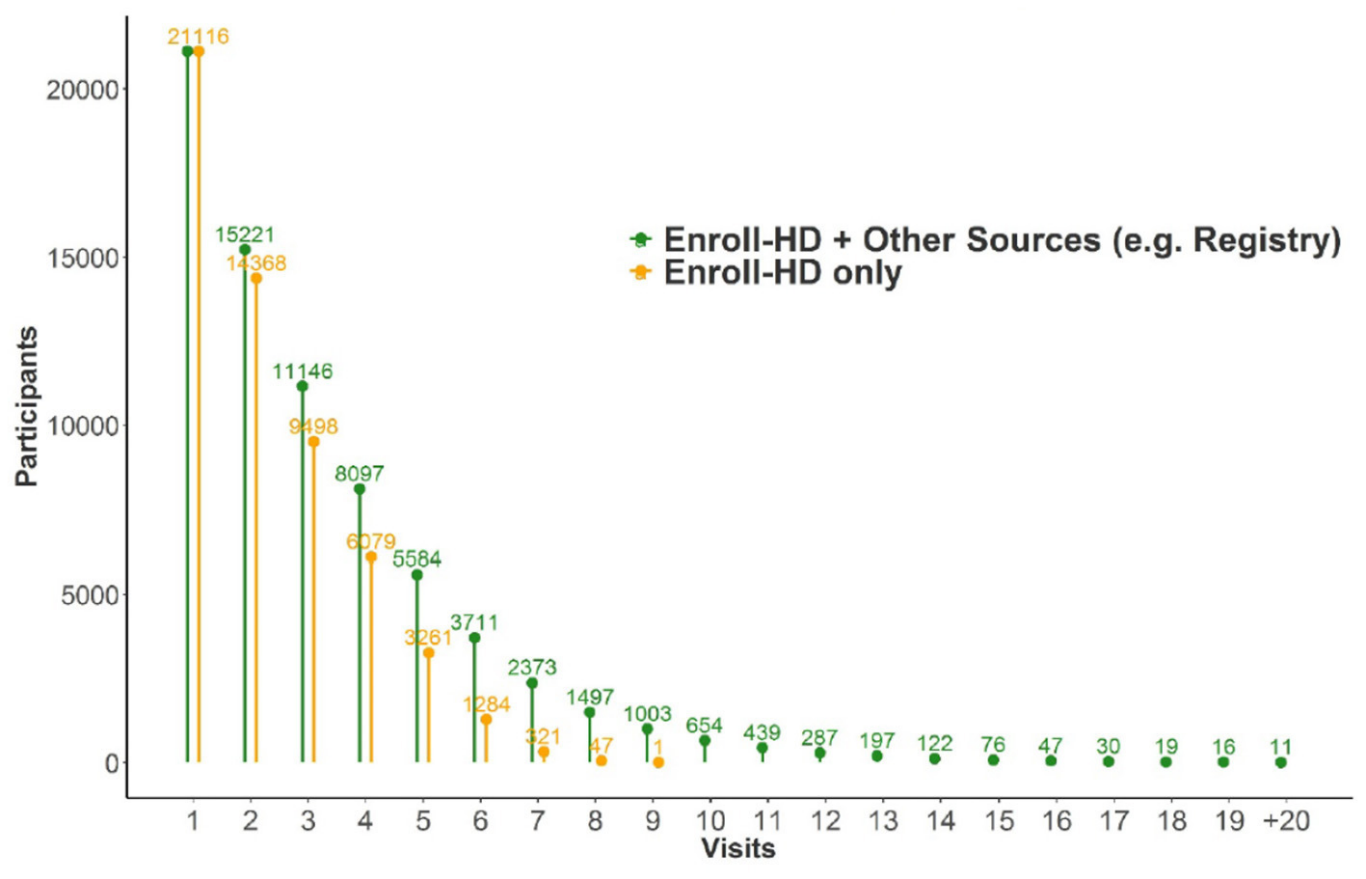
Number of participants from Enroll-HD alone and in combination with precursor studies like REGISTRY with specified number of visits (*N* = 21,116).

**Figure 3:**
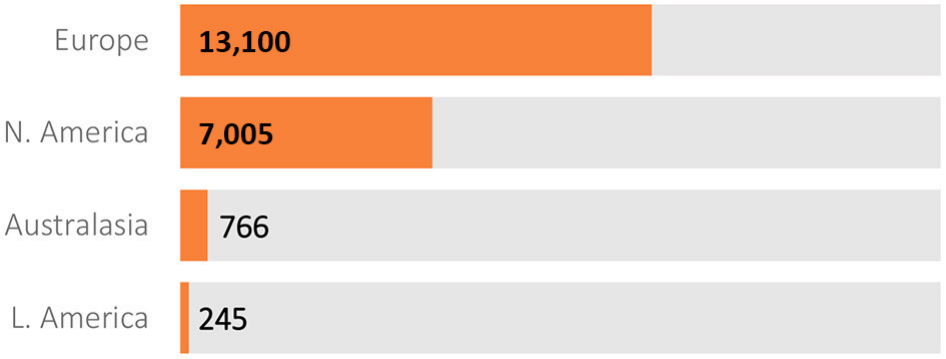
Geographical distribution of Enroll-HD participants (*N* = 21,116).

**Figure 4:**
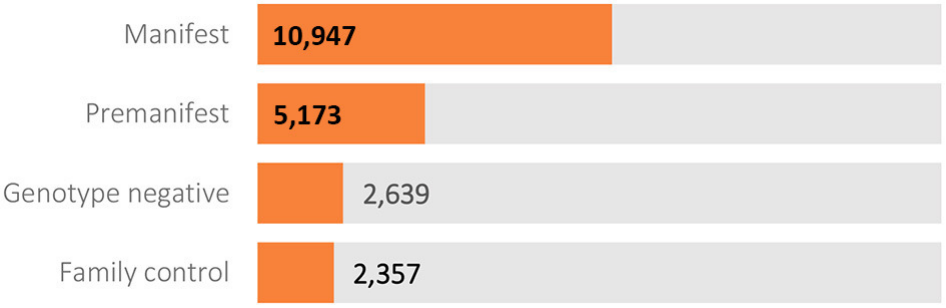
Participant category at Enroll-HD baseline visit (*N* = 21,116).

Data extracted from the database in October 2020 was released in December 2020 as periodic dataset 5 (PDS5; details below). This dataset contains data on 21,116 Enroll-HD participants (16,120 HDGECs and 4,996 non-HDGECs) from 71,682 visits (baseline and follow-up visits); 55,975 of these were Enroll-HD visits, with the remainder from REGISTRY (*N* = 14,737) and *ad hoc* sources such as unscheduled visits (*N* = 970). Participant sociodemographic distribution and disease characteristics (Table 3), the longitudinal data available (Figure 2), and the geographic distribution (Figure 3) and HD category distribution at baseline (Figure 4) of the Enroll-HD cohort are summarized. A detailed overview of the Enroll-HD PDS5 dataset is available at https://enroll-hd.org/for-researchers/technical-support/.

**Table 3:** Enroll-HD participant characteristics - sociodemographic and basic disease variables (PDS5 dataset; release 2020-10-31v1).

	**Total participants**	**HDGECs**	**Controls**
Number of participants	21,116	16,120	4,996
Sex, female; *N* (%)	11,783 (56%)	8,727 (54%)	3,056 (61%)
Age at baseline; mean (SD)	47.9 (14.0)	48.7 (14.0)	46.7 (14.8)
Education level (ISCED^a^; 0–3); *N* (%)	10,119 (48.1%)	8,130 (50.7%)	1,989 (40.0%)
Mean CAG length (SD)	N/A	43.6 (4.1)	20.2 (3.6)
CAP score^b^ at baseline; mean (SD)	N/A	97.2 (24.0)	N/A

a *ISCED education level dichotomized into binary variable: 0–3 and 4–6. Precise definitions for these categories vary by country. In the UK, 0–3 captures everything up to and including sixth form (i.e., further education), 4–6 captures university and beyond (i.e., higher education)*.

b *CAP score calculated using the formula CAP = AGE × (CAG−30)/6.49, which is standardized such that CAP = 100 at estimated age of disease onset*.

## Enroll-HD Supported Studies

Studies that use Enroll-HD platform services—clinical support and/or infrastructure services—are referred to as supported studies. Studies built into the platform and that recruit participants within the large Enroll-HD cohort (utilizing their annual-visit data and the extensive network of sites and investigators) and that involve additional assessments are referred to as nested studies; currently, these studies are investigating biomarkers and clinical or patient-reported outcomes. These arrangements reduce participant burden while allowing biomarker and outcome-related data to be linked. Ongoing and proposed supported and nested studies included below.

### HDClarity

HDClarity (NCT02855476) began in 2016 as a prospective nested annual CSF and blood (serum and plasma) collection initiative recruiting HDGECs and control participants from Enroll-HD. The large longitudinal cohort and the comprehensive phenotypic data linked to each CSF biosample are advantageous in validating potential biofluid biomarkers that were previously assessed in cross-sectional or longitudinal studies (18, 19), and to date more than 600 participants have donated CSF and blood biosamples at 24 study sites.

### ImageClarity

ImageClarity is a proposed nested study that will recruit eligible HDClarity participants to undergo an annual multisequence structural and functional modality brain magnetic resonance imaging (MRI). The goal is to expedite identification of biomarkers relevant across the full spectrum of pathological events, especially those occurring in very early stage HD, to evaluate disease progression in interventional trials.

### iMagemHTT

iMagemHTT (11C) (NCT03810898) is an ongoing nested PET-imaging study evaluating the binding and kinetic properties of the radioligand [^11^C]CHDI-180R and its suitability to measure aggregated mutant HTT in the brain, especially the basal ganglia, of HDGECs compared to non-HDGEC controls. This is an adaptive study that includes five go/no-go decision points dependent on the radioligand's promise that is being conducted at three sites in Belgium and the Netherlands.

iMagemHTT (F-18) is a proposed nested study currently in late-stage preclinical development that will comparatively evaluate multiple ^18^F-labeled, next-generation mutant HTT-targeting PET tracers. This first-in-human study will be conducted at a single site at Johns Hopkins University.

### Origin-HD

Origin-HD is a proposed nested cross-sectional, multi-site, observational study to investigate differences in germline and somatic *HTT* CAG-repeat instability and identify genetic modifiers of intergenerational CAG instability. Semen and blood biosamples from at least 1,000 male HDGECs will be collected at a single visit at around 40–50 sites.

### Later Stage HD Assessments

LSA is a nested study developing two assessments to measure critical milestones during advanced HD when travel is especially burdensome to participants; importantly, these assessments need to be conducted with the assistance of a participant companion, either in-person or remotely. The two-part study will evaluate the internal consistency, reliability, validity and clinimetric properties of the two assessments in development, initially at four sites and subsequently at 20 sites in the US and UK.

### FOCUS-HD

The proposed FOCUS-HD nested study aims to longitudinally validate FuRST 2.0—a patient reported outcome assessment sensitive to early functional changes in premanifest HDGECs—and HD-CAB—the only available fit-for-purpose HD cognitive assessment battery (20)—to define effect-size estimates of cognitive decline for power analyses in interventional trials. Cognitive pretesting for FuRST 2.0 was conducted at four Enroll-HD sites with eligible candidates and required resources.

### PACE-HD

PACE-HD (Physical Activity and Exercise Outcomes in Huntington's Disease; NCT03344601) is an ongoing interventional supported study conducted in about 120 HDGEC participants (21) that uses several aspects of the Enroll-HD platform—including core assessment data, onsite monitoring, and participant eligibility—to significantly reduce participant and site burden and speed study start-up by simplifying EDC system development.

## Enroll-HD Data and Biosample Availability

To accelerate HD therapeutic research and development, the platform provides any verified researcher access to the high-quality datasets and biosamples from Enroll-HD and nested studies. Enroll-HD clinical data are shared with the research community through periodic datasets (PDS) and specified datasets (SPS). PDSs are prepared from the Enroll-HD study database every 1–2 years and include a large majority of the collected variables. Prior to release each PDS undergoes stringent QC and coding procedures during which certain variables are transformed, aggregated, or suppressed (excluded) to protect participant identity. Access to non-transformed, non-aggregated, or suppressed data may be obtained through SPS request, subject to approval by the Scientific Review Committee (SRC) that weighs the scientific merit of the proposed project against the increased risk for participant identification. Renewable biosamples (lymphoblastoid cell lines, and DNA from such) can be obtained without review but non-renewable biosamples from Enroll-HD (DNA from whole blood, peripheral blood monocytes, EDTA plasma) or HDClarity (LiHep plasma, serum, CSF, cells from CSF) require merit review by the SRC.

Any researcher employed by a recognized research organization can open an Enroll-HD access account to obtain data and biosamples from Enroll-HD (or nested studies that make such resources available), subject to the researcher's employer/institution signing the appropriate data/biosample use agreement(s). Datasets with online click-through agreements, such as PDSs, are accessible immediately, whereas SPS and non-renewable biosample requests require SRC approval. All biosample requests require completion of a material transfer agreement (MTA) with wet ink signature. More detailed access information is available at https://enroll-hd.org/for-researchers/access-data/.

## Research using Enroll-HD data and biosamples

Enroll-HD datasets and biosamples have been used by researchers in academia, industry, and healthcare around the world to advance HD research. Enroll-HD PDSs have been downloaded 632 times and 124 SPS requests fulfilled. Further details on data users and their projects can be found at https://enroll-hd.org/for-researchers/current-enroll-hd-data-projects/, and a list of the publications using Enroll-HD data and/or biosamples is at https://enroll-hd.org/for-researchers/scientific-publications/. A series of articles providing useful advice on Enroll-HD data analysis is at https://enroll-hd.org/analysis-tools/.

Genome-wide association studies (GWAS) have already been conducted using biosamples from over 9,000 (soon to be 14,000) HDGEC participants, most of them from Enroll-HD as well as other HD clinical studies (22, 23). Using the difference between the CAG-length-predicted and actual age at motor onset as the predictor, three modifier signals at two loci within the *FAN1* and *MLH1* genes (involved in DNA maintenance and mitochondrial regulation) were found that affect motor onset. Drug development programs investigating genes involved in DNA mismatch repair are now underway.

Predictive and causal modeling for predictors of HD progression has also been undertaken using Enroll-HD data. Given the large number of healthy controls it was possible to establish cognitive and motor norms to determine the effects of natural aging and contrast these with HD progression. Normative curves by age, sex, and education have been estimated for the 0.05, 0.25, 0.50, 0.75, and 0.95 quantiles from the distribution of observed cognitive, motor and functional scores. Extreme quantile estimates for each measure can be considered as boundaries for natural aging, outside which can be attributed to HD pathology (24). Propensity score weighting to examine the effects of educational level, employment status, and tobacco, alcohol, recreational and prescription drug use on HD progression within the Enroll-HD cohort found that light and moderate alcohol use were not significantly linked to HD progression (25), contrary to a previous report (26). However, participants treated with antidepressants were likely to progress faster than non-users (27). A probabilistic machine learning-driven disease progression model that quantitatively describes complex changes around the time of clinical diagnosis has recently been developed; it has identified nine disease states within HD and can estimate the anticipated duration of each state (28). Further ongoing work to determine the likelihood that a participant will transition to the next state within a certain time period may assist in participant stratification to improve clinical trial design.

## Enroll-HD Platform Services

Scientific and operational support services are available to industrial and academic sponsors of HD interventional clinical trials and research studies. The Clinical Trial Committee (CTC) provides sponsors an opportunity to consult with highly experienced HD clinicians and researchers through all stages of protocol development regarding study design, endpoint strategy, and study population. CTC members review the study design, including the proposed assessments and endpoints, and ensure that participant interests are protected. Provided the protocol is rational and reasonable, the CTC issues a letter of acceptance that the sponsor can present to IRBs/ERBs.

The CTC also manages the HD Clinical Trial Site Certification program that uses an industry-standard framework to determine whether sites have the appropriate infrastructure to support clinical trials. Sites that are not part of Enroll-HD can apply for site certification to enable participation in other HD-related studies.

The Enroll-HD operational infrastructure also offers trial/study start-up and conduct to sponsors, with site feasibility assessment, country and site selection, eligible participant availability (in silico feasibility assessment), and liaison with local IRB/ERBs if requested.

## Enroll-HD Operational Infrastructure

Enroll-HD operates under a single study protocol worldwide and is supported by a comprehensive operational and oversight management infrastructure. Data quality and integrity is fundamental, and quality control and assurance measures designed to maximize data consistency, completeness, and accuracy are implemented and monitored at the participant, site, and study level. Enroll-HD's governance structure includes the CTC and SRC (see above), the Scientific Oversight Committee—which provides overall scientific strategy, ensures adherence to study and platform goals, and reviews non-interventional study protocols—and the Data Safety Monitoring Committee that reviews reportable events and addresses safety concerns. Together these committees ensure effective oversight of the platform and guidance to study site investigators and staff, regulatory authorities, researchers, and trial/study sponsors.

### Centralized EDC System

Enroll-HD uses a centralized multi-study electronic data capture (EDC) system that hosts Enroll-HD and supported/nested study data, providing a common data collection and reporting framework for every study site. This ensures the format and definitions of data entered are consistent on an intra- and inter-site level, cross-sectionally and longitudinally, in turn facilitating the application of centralized data QC procedures. System prompts, guidance documents, automated data validity checks, and automated field completion built into the EDC help ensure that accurate information is entered.

### Data Monitoring and Site Management

Remote centralized statistical monitoring (CSM) of data is conducted at the participant and site level by the data monitoring team. Participant-level data are subject to remote QC that checks cross-sectionally and longitudinally for consistency, completeness, and plausibility. Site-level CSM of compliance, performance, and data quality involves outlier analyses targeting site-specific operational performance metrics and clinical data, which may result in follow-up action (communication and training) with sites. All Enroll-HD sites are routinely monitored to review source data and ensure compliance with the study protocol, Good Clinical Practice (GCP), and other applicable regulations.

Sites also receive quarterly bulletins, semi-annual site metrics cards, and other communications. Tailored feedback and support are provided to improve underperforming sites and corrective action plans implemented when appropriate.

### Training

Investigators undergo standardized site-initiation training regarding the protocol, data entry and informed consent. To ensure consistency and accuracy in data collection, assessment-specific rater training and online certification is conducted through the Enroll-HD Clinical Training Portal (UHDRS motor certification and GCP), interactive webinar training (PBA-s), *ad hoc* training (in response to site-specific training needs), and self-study of online training materials. Onsite monitors also undergo standardized training to ensure consistency.

### Informed Consent and Participant Confidentiality

Recoded participant-level data and biosamples from Enroll-HD are made available to the research community under three stipulations: data and biosamples are shared subject to participants' informed consent and in accordance with GDPR (EU) and HIPAA (US) laws; a data use agreement (DUA) and/or material transfer agreement (MTA) must be signed and adhered to by any requester; and the risk of identification from their clinical data is assessed for all participants before data release and remedial steps taken for those above a predetermined risk threshold.

Participant identification recoding uses two data safety methods: the safe harbor method that removes 18 variables that may directly identify individuals (e.g., birth date, visit date, and site location), and the expert determination method where statisticians assess all individuals' risk of identification.

### Engagement With HD Advocacy Organizations

Enroll-HD works closely with HD patient advocacy organizations to engage their constituents in ongoing clinical research and provide updates on new developments in Enroll-HD. *Enroll!*, the Enroll-HD newsletter, features articles about ongoing HD research and clinical trials/studies, including Enroll-HD platform studies, as well as human interest stories from various stakeholders, and is translated into all the languages within Enroll-HD. The Enroll-HD platform also functions as a hub that enables collaborative projects with patient advocacy organizations.

## Discussion

Since its launch 9 years ago, Enroll-HD has developed into a multi-faceted clinical research platform that can support various clinical studies and interventional trials and has recruited almost 25,000 participants into the core worldwide longitudinal observational study, amassing a rich clinical database that is made available to any researcher. Enroll-HD has successfully incorporated all the required functionalities and provides a dynamic environment that supports clinical research to identify and develop biomarkers and clinically-relevant endpoints, validate novel patient-reported outcome measures, facilitate clinical trial recruitment and site feasibility, and provide expert scientific input on trial/study protocols. Enroll-HD is meeting its remit to better enable the clinical research needs required to develop HD therapeutics.

In addition to being a clinical research platform and observational study, Enroll-HD is also a patient registry, defined as “an organized system that uses observational study methods to collect uniform data (clinical and other) to evaluate specified outcomes for a population defined by a particular disease, condition or exposure, and that serves a predetermined scientific, clinical or policy purpose” (29). Such registries, particularly those for rare diseases, are especially useful when they make the collected longitudinal data widely available for research purposes, including to assess disease course (30).

In recent years there has been a drive to recruit more premanifest individuals, particularly young ones, into Enroll-HD to further understand this phase of the disease in preparation for clinical trials in, and subsequent treatment of, such individuals as early as possible. CHDI is now actively planning a companion study, SelfEnroll, that will remotely monitor premanifest participants' disease continuously rather than once a year.

Despite the demonstrated commitment of academic researchers, funding agencies, pharmaceutical companies, and patient advocacy groups, drug development for rare disorders has been hindered by sequestered research with insufficient collaboration (31) that often leads to duplicative and uncoordinated work. Enroll-HD is a cohesive endeavor to consolidate HD clinical research—thereby minimizing costs and patient burden while maximizing collaboration—that is designed to initiate, support and maintain additional studies planned by CHDI or other industrial/academic sponsors that can capitalize on the assembled research infrastructure, including the centralized EDC system, study site and participant selection, feasibility assessments, online training portal, standardized operating procedures, and access to a comprehensive network of HD clinicians and researchers.

## Author Contributions

SS and CS initially developed the manuscript concept and outline. SS wrote the first draft. JW, JL, and EN wrote sections of the manuscript. SN edited the manuscript throughout and added some further sections. All authors contributed to manuscript revision and have read and approved the submitted version.

## Conflict of Interest

The authors declare that the research was conducted in the absence of any commercial or financial relationships that could be construed as a potential conflict of interest.

## Publisher's Note

All claims expressed in this article are solely those of the authors and do not necessarily represent those of their affiliated organizations, or those of the publisher, the editors and the reviewers. Any product that may be evaluated in this article, or claim that may be made by its manufacturer, is not guaranteed or endorsed by the publisher.
